# Incidence and Resistance Patterns of *Citrobacter* spp. in Switzerland: A Nationwide, Retrospective Surveillance Study (2010–2022)

**DOI:** 10.3390/microorganisms13040786

**Published:** 2025-03-29

**Authors:** Pérince Fonton, Rebecca Grant, Michael Gasser, Niccolò Buetti, Andreas Kronenberg, Stephan Harbarth

**Affiliations:** 1 Infection Control Program, Geneva University Hospitals and Faculty of Medicine, WHO Collaborating Center, Rue Gabrielle-Perret-Gentil 4, CH-1205 Geneva, Switzerland; perince.fonton@hug.ch (P.F.); rebecca.grant@hug.ch (R.G.); niccolo.buetti@hug.ch (N.B.); 2 Swiss Centre for Antibiotic Resistance (ANRESIS), Institute for Infectious Diseases, University of Bern, 3001 Bern, Switzerland; michael.gasser@unibe.ch (M.G.); andreas.kronenberg@unibe.ch (A.K.)

**Keywords:** *Citrobacter* spp., antimicrobial resistance, bloodstream infections, urinary tract infections, Switzerland, incidence, epidemiological surveillance

## Abstract

We conducted a retrospective analysis of *Citrobacter* spp. surveillance data from acute care hospitals that contributed *Citrobacter* spp. data to the national surveillance system ANRESIS from January 2010 to December 2022. The incidence of *Citrobacter* spp. bloodstream infections (BSIs) in Switzerland was calculated, as well as the proportion of *Citrobacter* spp. isolates from urinary tract samples. We also evaluated the susceptibility of *Citrobacter* spp. isolates to clinically important antibiotics. From 2010 to 2022, there were 33,958 *Citrobacter* spp. from patients across 55 acute care hospitals continuously participating in ANRESIS included in this analysis. We observed an annual increase in the number of *Citrobacter* spp. BSIs, from 2.5 to 4.2 cases per 100,000 patient days (IRR: 1.04, 95% CI: 0.96–1.12). We found a higher incidence among male versus female patients (IRR: 2.47, 95% CI: 1.28–4.74) and in those aged ≥65 years, as compared with younger patients (IRR: 2.26, 95% CI: 1.18–4.32). The proportion of *Citrobacter* spp. among positive urinary tract samples also increased (from 18.6 to 24.7 per 1000 samples). Among ICU patients, there was a considerable proportion of resistance to third-generation cephalosporins among *C. freundii* isolates (26.8–44.0%), compared with non-*freundii* isolates (1.7–6.9%). *Citrobacter* spp. is gaining clinical importance in Switzerland; further studies are needed to better understand the underlying mechanisms.

## 1. Introduction

*Citrobacter* spp., mainly *C. freundii*, are increasingly recognized as primarily healthcare-associated pathogens causing urinary, abdominal, and respiratory tract infections [1,2]. A recent systematic review of 41 studies found that approximately 85% of *Citrobacter* spp. infections were hospital-acquired [2]. This review highlighted an upward trend in the incidence of *Citrobacter* infections since 2010, underscoring the growing challenge these pathogens present within healthcare settings [2].

Further, over the past five years, surveillance studies conducted across several European countries, including Germany, Spain, and Finland, have documented an increase in *Citrobacter* isolates with clinically relevant resistance genes, including extended-spectrum β-lactamases (ESBLs), *Klebsiella pneumoniae* carbapenemase (KPC), metallo-β-lactamases (e.g., VIM), and OXA-48 carbapenemases [1,3,4]. Among *Citrobacter* species, *C. freundii* may present particular therapeutic challenges through resistance to multiple antibiotics [5].

There is currently limited information available on the epidemiological burden, temporal trends, and resistance patterns of *Citrobacter* spp. The objective of this study was to describe the clinical and epidemiological features of *Citrobacter* spp. among hospitalized patients in Switzerland using national surveillance data generated by the Swiss National Centre for Antibiotic Resistance (ANRESIS), which collects routine antibiotic resistance data from microbiology laboratories located across Switzerland [6].

## 2. Materials and Methods

### 2.1. Study Design and Inclusion Criteria

We conducted a retrospective analysis of *Citrobacter* spp. reported to ANRESIS from 1 January 2010 to 31 December 2022. During this period, acute care hospitals contributing data to ANRESIS represented an average of 72% of annual patient days across Switzerland. As the number of acute care hospitals participating in ANRESIS increased during the study period, we restricted the present analysis to data from acute care hospitals that reported at least one *Citrobacter* spp. to ANRESIS each year during the entire 13-year study period. We included only the first *Citrobacter* spp. isolate per patient per year reported to ANRESIS.

### 2.2. Data Sources

Participating hospitals are requested to report to ANRESIS all *Citrobacter* isolates. Patient-level data reported to ANRESIS included demographic information, specimen collection year and type (blood culture, urinary tract, respiratory tract, gastrointestinal tract, or other), location of patient consultation (inpatient versus outpatient), and type of unit of hospitalization (intensive care unit (ICU) versus non-ICU). The following institutional-level information was also collected: linguistic region of Switzerland (German-speaking versus Latin-speaking, which combined French and Italian-speaking cantons) and type of hospital (university vs. non-university). For each *Citrobacter* spp. isolate phenotypic antimicrobial susceptibility data were also included.

For antimicrobial susceptibility testing, isolates were considered susceptible (susceptible or susceptible, increased exposure), resistant, or unknown when the antimicrobial susceptibility testing had not been performed. In each of the selected laboratories, antimicrobial susceptibility testing was performed according to the guidelines of the Clinical and Laboratory Standards Institute (CLSI) or the European Committee on Antimicrobial Susceptibility Testing (EUCAST) [7,8]. Most laboratories switched from CLSI to EUCAST breakpoints between 2011 and 2013. We assessed *Citrobacter* spp. susceptibility to clinically important antibiotics, including cefepime, third-generation cephalosporins, carbapenems, and fluoroquinolones among patients in three settings: (1) ICU, (2) non-ICU inpatient wards, and (3) outpatient departments. The results were stratified by *Citrobacter* species: *C. freundii* versus non-*freundii*. A *Citrobacter* spp. isolate was considered resistant to an antibiotic group if resistant to at least one antibiotic within a given group (cefepime, third-generation cephalosporins, carbapenems, and fluoroquinolones).

### 2.3. Statistical Analysis

We described the characteristics of *Citrobacter* spp. included in the analysis. The categorical variables were reported as frequencies and proportions. Differences in the characteristics of patients by *Citrobacter* species were compared using Pearson’s chi-squared test. The incidence of *Citrobacter* spp. from blood cultures in Switzerland and per linguistic region was calculated per 100,000 patient days using annual patient-day data for each acute care hospital included in the analysis from the Federal Office of Public Health (FOPH) [9]. The results were then stratified by *Citrobacter* species, sex, and age category (<18 years, 18–64 years, ≥65 years). We modeled temporal trends in the incidence of *Citrobacter* spp. bloodstream isolates using negative binomial regression with a log-link function, accounting for overdispersion, and included patient days as an offset variable. The model included year, linguistic region, sex, and age category (<18 years, 18–64 years, ≥65 years) as covariates.

We calculated the proportion of *Citrobacter* spp. in urinary tract samples across Switzerland and by linguistic region, expressed per 1000 positive urinary tract samples (all bacterial species) submitted to ANRESIS. This approach accounts for annual variations in the total number of urinary tract samples sent to ANRESIS. As above, the results were stratified by *Citrobacter* species, sex, and age category (<18 years, 18–64 years, and ≥65 years). For all analyses, *p*-values ≤ 0.05 were considered statistically significant. All statistical analyses were performed with R statistical software version 3.5.2 (R Foundation, Vienna, Austria).

## 3. Results

### 3.1. Characteristics of Citrobacter spp. Species

**Figure 1:**
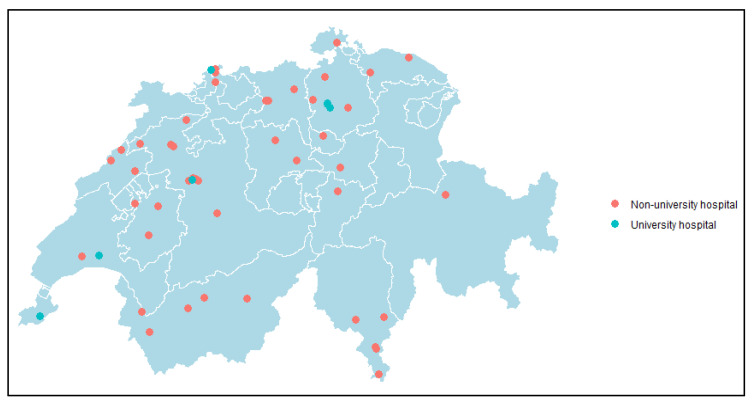
Map of Switzerland indicating the 55 included acute care hospitals that reported at least one *Citrobacter* spp. to ANRESIS each year from 1 January 2010 to 31 December 2022. Green circles represent university hospitals; orange circles represent non-university hospitals.

Between 1 January 2010 and 31 December 2022, 33,958 *Citrobacter* spp. were reported to ANRESIS from 55 acute care hospitals across Switzerland (Figure 1). Characteristics of *Citrobacter* spp. are presented in Table 1. Among *Citrobacter* species, the majority were *C. koseri* (50.6%), followed by *C. freundii* species (39.9%).

### 3.2. Citrobacter spp. Bloodstream Infections (BSIs)

**Figure 2:**
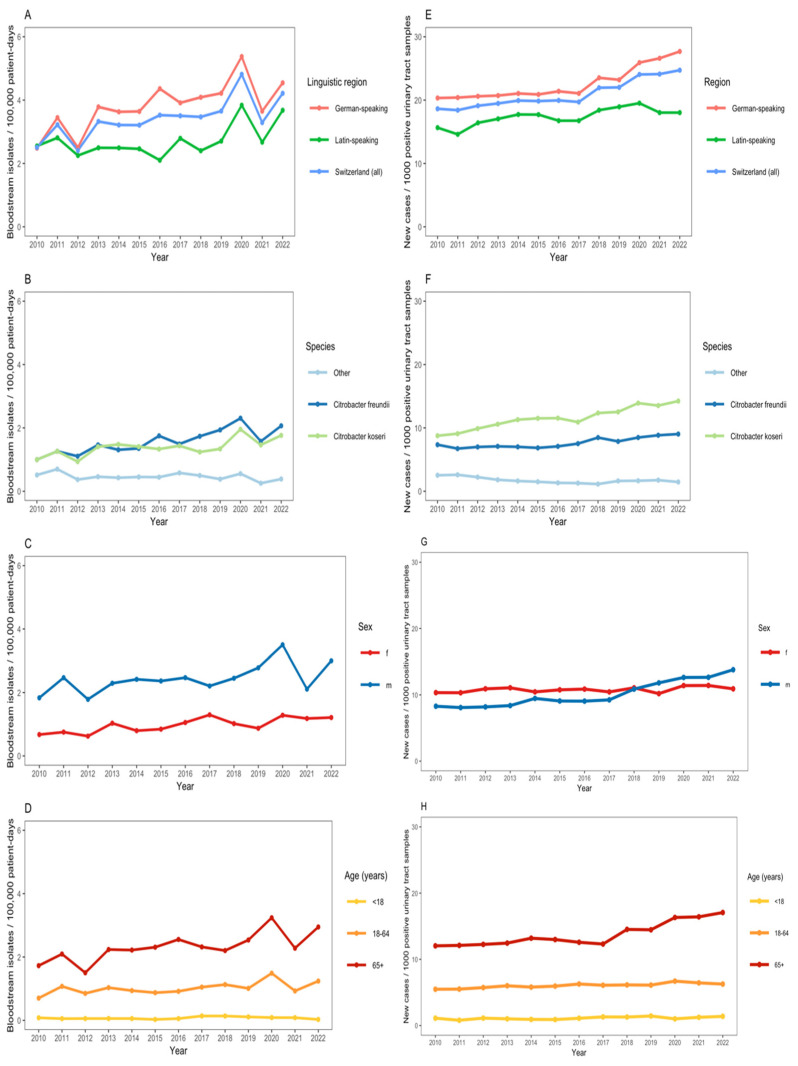
(**A**–**D**): Incidence of *Citrobacter* bacteremia per 100,000 patient-days across 55 ANRESIS acute care hospitals in Switzerland, 2010–2022, N = 1590, stratified by linguistic region (**A**), *Citrobacter* species (**B**), sex (**C**), and age group (**D**). (**E**–**H**): Proportion of *Citrobacter* spp. from urinary tract samples per 1000 positive urinary tract samples sent to ANRESIS across 55 acute care hospitals in Switzerland, 2010–2022, N = 19,699, stratified by linguistic region (**E**), *Citrobacter* species (**F**), sex (**G**) and age group (**H**).

We observed an increase in the annual number of *Citrobacter* spp. BSIs (from 2.5 cases per 100,000 patient days in 2010 to 4.2 cases in 2022) with a notable increase during the first pandemic COVID-19 waves in 2020 (Figure 2A). *C. koseri* and *C. freundii* were the predominant *Citrobacter* species among bloodstream isolates (Figure 2B). The incidence of *Citrobacter* spp. BSI was greater in males, as compared with females, and in patients aged ≥65 years (Figure 2C,D). The adjusted negative binomial regression model indicated a non-significant annual increase in the incidence of *Citrobacter* spp. BSIs of 3.7% (IRR: 1.04, 95% CI: 0.96–1.12). In the same adjusted model, the incidence rate of *Citrobacter* spp. BSIs in the German-speaking region was 2.41 (95% CI: 1.26–4.61) as compared with the Latin-speaking region; males had more than twice the incidence rate of *Citrobacter* spp. BSIs compared with females (IRR: 2.47, 95% CI: 1.28–4.74); individuals aged ≥65 years had a significantly higher incidence rate of BSIs compared with the 18–64 age group (IRR: 2.26, 95% CI: 1.18–4.32).

### 3.3. Citrobacter spp. from Urinary Tract Samples

There was also an increase in the annual proportion of *Citrobacter* spp. per 1000 positive urinary tract samples submitted to ANRESIS (from 18.6 per 1000 positive urinary tract samples in 2010 to 24.7 in 2022) (Figure 2E). The annual proportion of *Citrobacter* spp. among positive urinary tract samples was greater in the German-speaking region of Switzerland than in the Latin-speaking region (Figure 2E). *C. koseri* and *C. freundii* were the predominant *Citrobacter* species among positive urinary tract samples (Figure 2F). From 2017 onwards, there was a notable increase in the proportion of *Citrobacter* spp. among male patients and in those ≥65 years (Figure 2G,H).

### 3.4. Citrobacter spp. Antibiotic Susceptibility

**Figure 3:**
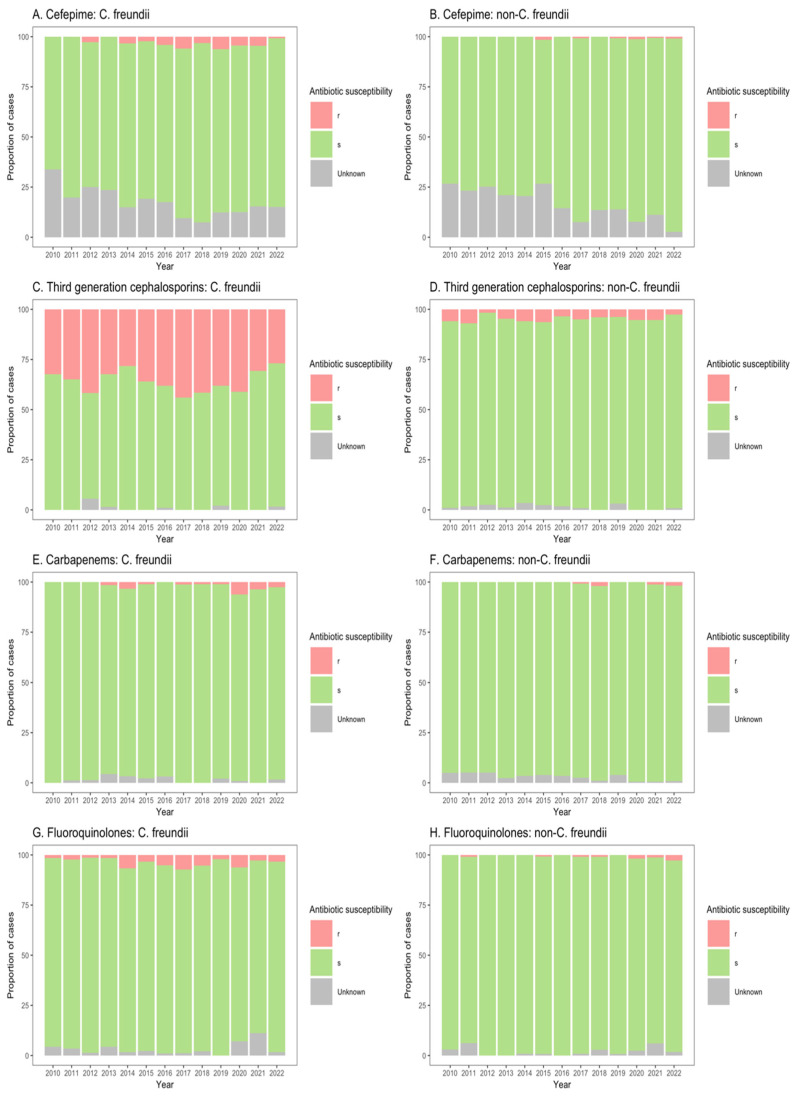
Susceptibility of *Citrobacter freundii* and non-*freundii* isolates to cefepime (**A**,**B**), third-generation cephalosporins (**C**,**D**), carbapenems (**E**,**F**), and fluoroquinolones (**G**,**H**) among patients admitted to intensive care units from 55 ANRESIS acute care hospitals in Switzerland, 2010–2022. Isolates were considered as ‘s’ (susceptible or susceptible, increased exposure), r’ (resistant), or ‘Unknown’.

**Figure 4:**
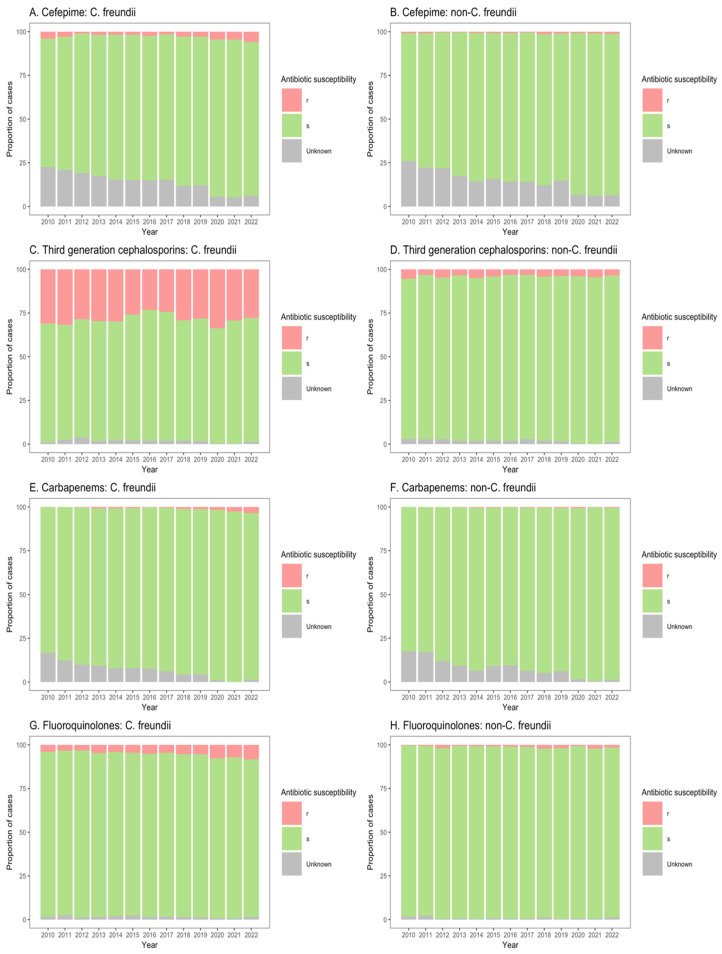
Susceptibility of *Citrobacter freundii* and non-*freundii* isolates to cefepime (**A**,**B**), third-generation cephalosporins (**C**,**D**), carbapenems (**E**,**F**), and fluoroquinolones (**G**,**H**) among patients in non-ICU inpatient wards across 55 ANRESIS acute care hospitals in Switzerland, 2010–2022. Isolates were considered as ‘s’ (susceptible or susceptible, increased exposure), r’ (resistant), or ‘Unknown’.

**Figure 5:**
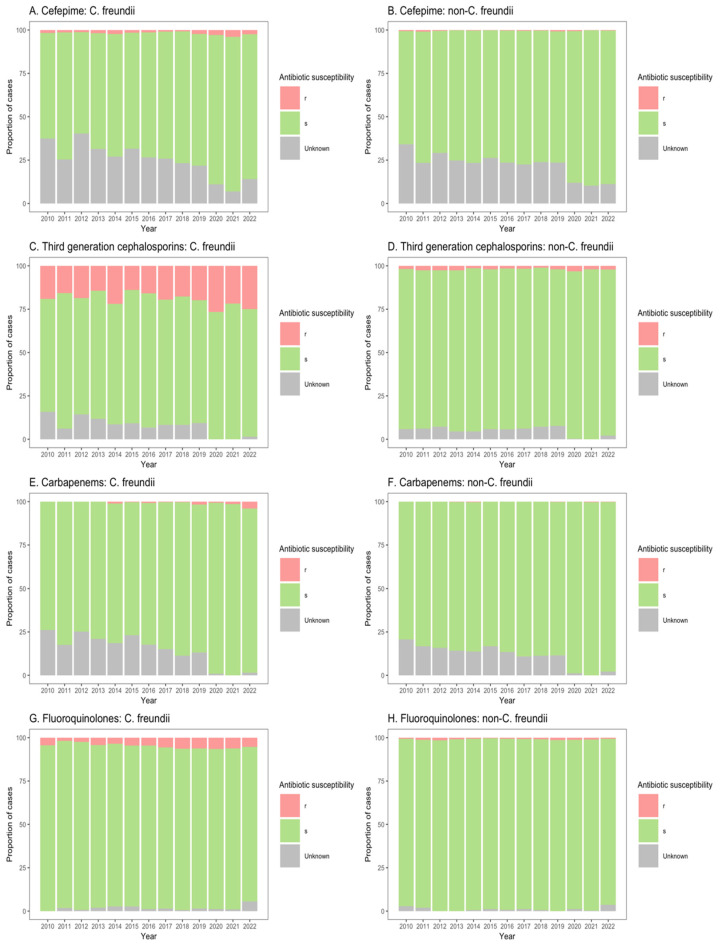
Susceptibility of *Citrobacter freundii* and non-*freundii* isolates to cefepime (**A**,**B**), third-generation cephalosporins (**C**,**D**), carbapenems (**E**,**F**), and fluoroquinolones (**G**,**H**) among outpatient departments across 55 ANRESIS acute care hospitals in Switzerland, 2010–2022. Isolates were considered as ‘s’ (susceptible or susceptible, increased exposure), r’ (resistant), or ‘Unknown’.

The susceptibilities of *Citrobacter* spp. isolates to clinically important antibiotics among patients from ICU, non-ICU inpatient wards, and outpatient departments are shown in Figure 3, Figure 4 and Figure 5. Among ICU patients, there was a considerable proportion of resistance to third-generation cephalosporins among *C. freundii* isolates (26.8–44.0%, Figure 3C), as compared with non-*freundii* isolates (1.7–6.9%, Figure 3D). *C. freundii* isolates also exhibited higher resistance rates to cefepime, carbapenems, and fluoroquinolones compared with non-*freundii* isolates among ICU patients (Figure 3).

Similarly, for patients in both non-ICU inpatient wards and outpatient departments, there was a considerable proportion of resistance to third-generation cephalosporins among *C. freundii* isolates (23.2–33.8% of isolates from non-ICU inpatients (Figure 4C); 13.9–26.6% of isolates from outpatients (Figure 5C)), as compared with non-*freundii* isolates (3.2–5.4% of isolates from non-ICU inpatients (Figure 4D); 1.1–3.1% of isolates from outpatient departments (Figure 5D)). *C. freundii* isolates also demonstrated greater proportions of resistance to cefepime and fluoroquinolones, as compared with non-*freundii* isolates, among patients in non-ICU inpatient wards and outpatient departments (Figure 4 and Figure 5). The proportion of isolates demonstrating carbapenem resistance remained low across all settings: 1.0–6.3% for *C. freundii* and 0.8–1.9% for non-*freundii* isolates from ICU patients (Figure 3E,F); 0.1–3.5% for *C. freundii* and 0.1–0.7% for non-*freundii* isolates from non-ICU inpatients (Figure 4E,F); 0.3–3.9% for *C. freundii* and 0.1–0.4% for non-*freundii* isolates from outpatients (Figure 5E,F).

## 4. Discussion

We used surveillance data from 55 Swiss acute care hospitals participating in ANRESIS to describe the clinical and epidemiological features of *Citrobacter* spp. over a period of 13 years. Our findings indicate an increase in the annual incidence of *Citrobacter* spp. BSIs. We found a higher incidence among male versus female patients, in those aged ≥65 years, as compared with younger patients, and those living in the German-speaking region of Switzerland, as compared with the Latin-speaking region. We also demonstrated an increase in the proportion of *Citrobacter* spp. among positive urinary tract samples, particularly from 2017 onwards among male patients and in those ≥65 years. These findings support our assertion that, although *Citrobacter* spp. have long been considered to be pathogens of low virulence, they are, in fact, emerging pathogens of clinical importance in Switzerland.

There are few studies on the burden and trends of *Citrobacter* spp. for comparative analysis. For example, *Citrobacter* spp. are not included in the European Antimicrobial Resistance Surveillance Network (EARS-Net) or WHO Global Antimicrobial Resistance and Use Surveillance System (GLASS) reporting. Nevertheless, a 10-year period surveillance study conducted in Hungary from 2008 to 2017 revealed an increase in urinary tract infections (UTIs) caused by *Citrobacter* spp. among inpatients and outpatients [10]. The study found that the most affected age groups were those under 10 years and over 60 years of age [10]. In the Czech Republic, from 2011 to 2019, Hrbacek et al. also reported an increase in the prevalence of *Citrobacter* spp. from positive urine samples [11]. A recent systematic review documented an increasing number of *Citrobacter* infections or colonization since 2010, particularly in Asian countries [2]. Further, several European surveillance studies have reported increasing rates of *Citrobacter* spp. isolates carrying extended-spectrum beta-lacatamase and carbapenemase genes [1,3,4]. We found a considerable proportion of *C. freundii* isolates demonstrating resistance to third-generation cephalosporins among patients in ICU, most likely based on AmpC β-lactamases [12,13]. In contrast, the proportion of isolates with carbapenem resistance remained very low in our study.

The main strength of this study is that it is based on a large national database of *Citrobacter* spp. from 55 acute care hospitals, which account for 72% of annual patient days across Switzerland. This study, therefore, offers an important contribution to otherwise sparse epidemiological data on *Citrobacter* spp. burden, temporal trends, and resistance patterns. Further, we restricted our analyses to account for an increase in the number of acute care hospitals contributing data to ANRESIS throughout the study period. Our analyses were also stratified by linguistic regions of Switzerland (German- versus Latin-speaking), reflecting the country’s linguistic and cultural heterogeneity. Our results from urinary tract samples highlight the need for further studies to explain the increase in the proportion of *Citrobacter* spp. among urinary tract samples, particularly among male patients and in those ≥65 years from 2017 onwards.

Nevertheless, there are a number of limitations. The ANRESIS database does not include clinical information or genotypic data. We were also unable to distinguish infection from colonization for non-invasive specimens, thereby affecting our assessment of the *Citrobacter* spp. infection burden beyond BSIs. Finally, we were not able to distinguish community-acquired from healthcare-associated infections, limiting our understanding of the role of nosocomial acquisition in the observed trends. However, the increase in *Citrobacter* spp. BSIs and the proportion of carbapenem resistance among ICU patients observed during the early COVID-19 pandemic could be an indirect indicator of related infection control challenges.

In conclusion, our study offers valuable insights into the epidemiology of *Citrobacter* spp. in Switzerland. The findings indicate that *Citrobacter* spp. are emerging as clinically important pathogens. Further epidemiological and molecular studies are needed to elucidate the mechanisms underlying these trends.

## Figures and Tables

**Table 1:** Characteristics of 33,958 *Citrobacter* spp. from 55 ANRESIS acute-care hospitals, 2010–2022.

		All *Citrobacter* Species (N = 33,958)		*C. koseri* (N = 17,173)		*C. freundii* (N = 13,540)		Other *Citrobacter* spp. (N = 3245)		*p*-Value
		n	(%)	n	(%)	n	(%)	n	(%)	
Age (years)	<18	1780	(5.2)	764	(4.4)	800	(5.9)	216	(6.7)	<0.01
	18–64	11,285	(33.2)	6307	(36.7)	4030	(30.0)	948	(29.2)	
	≥65	20,893	(61.5)	10,102	(58.8)	8710	(64.0)	2081	(64.1)	
Sex	Female	15,043	(44.0)	6456	(38.0)	6891	(51.0)	1696	(52.0)	<0.01
	Male	18,908	(56.0)	10,714	(62.0)	6645	(49.0)	1549	(48.0)	
	Unknown	7		3		4		0		
Linguistic region *	German-speaking	24,813	(73.0)	12,559	(73.0)	10,123	(75.0)	2131	(66.0)	<0.01
	Latin-speaking	9145	(27.0)	4614	(27.0)	3417	(25.0)	1114	(34.0)	
Hospital type	University hospital	14,015	(41.0)	7297	(42.0)	5703	(42.0)	1015	(31.0)	<0.01
	Non-university hospital	19,943	(59.0)	9876	(58.0)	7837	(58.0)	2230	(69.0)	
Location of consultation	Inpatient	23,593	(69.0)	11,286	(66.0)	9901	(73.0)	2406	(74.0)	<0.01
	Outpatient	10,365	(31.0)	5887	(34.0)	3639	(27.0)	839	(26.0)	
Unit of hospitalization	ICU admission	2433/23,593	(10.3)	1136/11,286	(10.1)	1048/9901	(10.6)	249/2406	(10.3)	0.5
	No ICU admission	21,160/23,593	(89.7)	10,150/11,286	(89.9)	8853/9901	(89.4)	2157/2406	(89.6)	
Sample type	Blood	1522	(4.5)	610	(3.6)	703	(5.2)	209	(6.4)	<0.01
	Gastrointestinal tract	1092	(3.2)	135	(0.8)	693	(5.1)	264	(8.1)	
	Respiratory tract	3917	(11.5)	1973	(11.5)	1695	(12.5)	249	(7.7)	
	Urinary tract	19,699	(58.0)	10,935	(63.7)	7185	(53.1)	1579	(48.7)	
	Other	7728	(22.8)	3520	(20.5)	3264	(24.1)	944	(29.1)	

* Latin-speaking region defined as the following cantons: Geneva, Neuchatel, Jura, Ticino, Vaud; all other cantons in Switzerland were considered as German-speaking.

## Data Availability

Data are provided within the manuscript. Further inquiries can be directed to the corresponding author.

## Abbreviations

The following abbreviations are used in this manuscript:

ANRESIS	Swiss National Centre for Antibiotic Resistance
BSI	Bloodstream infection
*C. freundii*	*Citrobacter freundii*
CI	Confidence interval
*C. koseri*	*Citrobacter koseri*
ICU	Intensive care unit
IRR	Incidence rate ratio
